# *PTCHD1* gene mutation/deletion: the cognitive-behavioral phenotyping of four case reports

**DOI:** 10.3389/fpsyt.2023.1327802

**Published:** 2024-01-15

**Authors:** Federica Alice Maria Montanaro, Alessandra Mandarino, Viola Alesi, Charles Schwartz, Daniela Judith Claps Sepulveda, Cindy Skinner, Michael Friez, Gabriele Piccolo, Antonio Novelli, Ginevra Zanni, Maria Lisa Dentici, Stefano Vicari, Paolo Alfieri

**Affiliations:** ^1^Child and Adolescent Neuropsychiatry Unit, Department of Neuroscience, Bambino Gesù Children’s Hospital, IRCCS, Rome, Italy; ^2^Laboratory of Medical Genetics, Translational Cytogenomics Research Unit, Bambino Gesù Children Hospital, IRCCS, Rome, Italy; ^3^Department of Pediatrics and Human Development, College of Human Medicine, Michigan State University, East Lansing, MI, United States; ^4^Neurology Unit, Bambino Gesù Children's Hospital, IRCCS, Rome, Italy; ^5^Greenwood Genetic Center, Gregor Mendel Circle, Greenwood, SC, United States; ^6^Unit of Muscular and Neurodegenerative Disorders, Unit of Developmental Neurology, Bambino Gesù Children’s Hospital, IRCCS, Rome, Italy; ^7^Genetics and Rare Diseases Research Division, Bambino Gesù Children's Hospital, IRCCS, Rome, Italy

**Keywords:** *PTCHD1* gene, intellectual disability, autism spectrum disorder, rare genetic syndrome, cognitive-behavioral phenotype

## Abstract

**Introduction:**

X-linked *PTCHD1* gene has recently been pointed as one of the most interesting candidates for involvement in neurodevelopmental disorders (NDs), such as intellectual disability (ID) and autism spectrum disorder (ASD). *PTCHD1* encodes the patched domain-containing protein 1 (*PTCHD1*), which is mainly expressed in the developing brain and adult brain tissues. To date, major studies have focused on the biological function of the *PTCHD1* gene, while the mechanisms underlying neuronal alterations and the cognitive-behavioral phenotype associated with mutations still remain unclear.

**Methods:**

With the aim of incorporating information on the clinical profile of affected individuals and enhancing the characterization of the genotype–phenotype correlation, in this study, we analyze the clinical features of four individuals (two children and two adults) in which array-CGH detected a *PTCHD1* deletion or in which panel for screening non-syndromal XLID (X-linked ID) detected a *PTCHD1* gene variant. We define the neuropsychological and psychopathological profiles, providing quantitative data from standardized evaluations. The assessment consisted of clinical observations, structured interviews, and parent/self-reported questionnaires.

**Results:**

Our descriptive analysis align with previous findings on the involvement of the *PTCHD1* gene in NDs. Specifically, our patients exhibited a clinical phenotype characterized by psychomotor developmental delay- ID of varying severity. Interestingly, while ID during early childhood was associated with autistic-like symptomatology, this interrelation was no longer observed in the adult subjects. Furthermore, our cohort did not display peculiar dysmorphic features, congenital abnormalities or comorbidity with epilepsy.

**Discussion:**

Our analysis shows that the psychopathological and behavioral comorbidities along with cognitive impairment interfere with development, therefore contributing to the severity of disability associated with *PTCHD1* gene mutation. Awareness of this profile by professionals and caregivers can promote prompt diagnosis as well as early cognitive and occupational enhancement interventions.

## 1 Introduction

Deletions in patched domain-containing 1 (*PTCHD1*) located at Xp22.1 have been linked to intellectual disability (ID) and autism spectrum disorder (ASD) (1–3). Both common and rare variants of *PTCHD1* have been reported to contribute to ASD and ID (4). For instance, a 27 bp duplication in the promoter region of *PTCHD1*, showing a significant decrease (26%) in the transcriptional activity of *PTCHD1*, was identified in three patients with ASD, while one missense variant [chrX.hg19. g.23353144G > A, p.(Ser51Asn)] was found in an ID patient (4). *PTCHD1* is a member of the patched domain-containing protein family, which also includes the Hedgehog (Hh) pathway receptor Patched 1 (PTCH1) and Niemann–Pick disease type C1 (NPC1). *PTCHD1* is an 888-amino acid, 12-pass transmembrane protein containing a sterol sensing domain (SSD) composed of TM 2–6 and a sterol sensing-like domain consisting of TM7-12 followed by a C terminal tail containing 52 terminal tail containing a PDZ-binding motif. Additionally, two large ectodomains (ECDs) are inserted between TM1 and TM2 and TM7 and TM8 (5).

Regarding its structural function, encodes for a transmembrane protein with a patched domain that was initially described in the Drosophila, which has the function of carrying sterols and lipids and works as a receptor for the sonic hedgehog (Hh) (6). Hh is one of the key signaling pathways involved in the formation of the neural tube and brain (7) and in the post-natal brain, it plays a key role in controlling neuronal precursor proliferation in the developing cerebellum and developing dentate granule cells and adult neural precursors. For this reason, while in the past it has been hypothesized as a possible contribution of the gene to the sonic hedgehog (Shh) signaling and synapse formation (8, 9), recent studies have demonstrated that, unlike PTCH1, *PTCHD1* ectodomains do not bind Shh but a series of RNA binding proteins involved in stress granule and ribonucleoprotein granule assembly (5).

To better understand the cellular and molecular function of the *PTCHD1* protein, Tora et al. (9) analyzed *PTCHD1* knock-out (KO) male mice, revealing its critical role in the excitatory/inhibitory balance in dentate granule cells, even though it is not required for structural synapse formation in the hippocampal dentate gyrus. Furthermore, a study investigating the autistic phenotype associated with deletions in *PTCHD1* AS (autism-associated) demonstrated how induced pluripotent stem cell-derived neurons from subjects with ASD and deletions of the *PTCHD1* locus exhibited reduced miniature excitatory postsynaptic current frequency and N-methyl-D-aspartate receptor hypofunction. This finding would suggest that these deletions may be involved in the neurophysiology of excitatory synapses and synaptic impairment associated with ASD (10, 11). Despite these major advances, few studies have already investigated the clinical features related to gene deletion or mutation. For instance, Filges et al. (2) described two brothers with X-linked inheritance with a deletion of *PTCHD1*, both presenting with ID. Interestingly, while the elder showed severe ID only, the youngest exhibited additional autistic features along with absent expressive speech, therefore suggesting a variable phenotype. Furthermore, Chaundhry et al. (1) reported phenotype descriptions of 23 individuals from 16 families with *PTCHD1* exonic deletions or truncating mutations, confirming that the hemizygous *PTCHD1* loss of function causes an X-linked neurodevelopmental disorder with a strong prevalence of autistic behaviors. Additionally, they found that individuals with *PTCHD1* disruption may exhibit motor disorders, a variable spectrum of ID, and other behavioral manifestations such as tics, anxiety, attention deficit and/or hyperactivity, impulsivity, aggressive behaviors, and sleep disorder. Notably, their study did not reveal the presence of congenital anomalies, epilepsy, or abnormal electroencephalography (EEG) pattern, although *PTCHD1* has been also proposed as a candidate epilepsy gene, by Rochtus et al. in a study analyzing the whole-exome sequencing of children with epilepsy (10). Furthermore, Wells et al. (12) underlined how *PTCHD1* is usually expressed in the thalamic reticular nucleus of mice in early development and how its loss determines attention deficit and hyperactivity behaviors, providing insight into the clinical manifestation observed in individuals with *PTCHD1* mutations.

With the aim to contribute to the cognitive-behavioral characterization of *PTCHD1* mutations, here, we describe four individuals with deletion or non-synonymous variants in the *PTCHD1* gene identified by array-CGH and by an X Linked Intellectual Disability (XLID) Panel, providing quantitative data from standardized evaluations.

## 2 Methods

### 2.1 Participants

**Figure 1:**
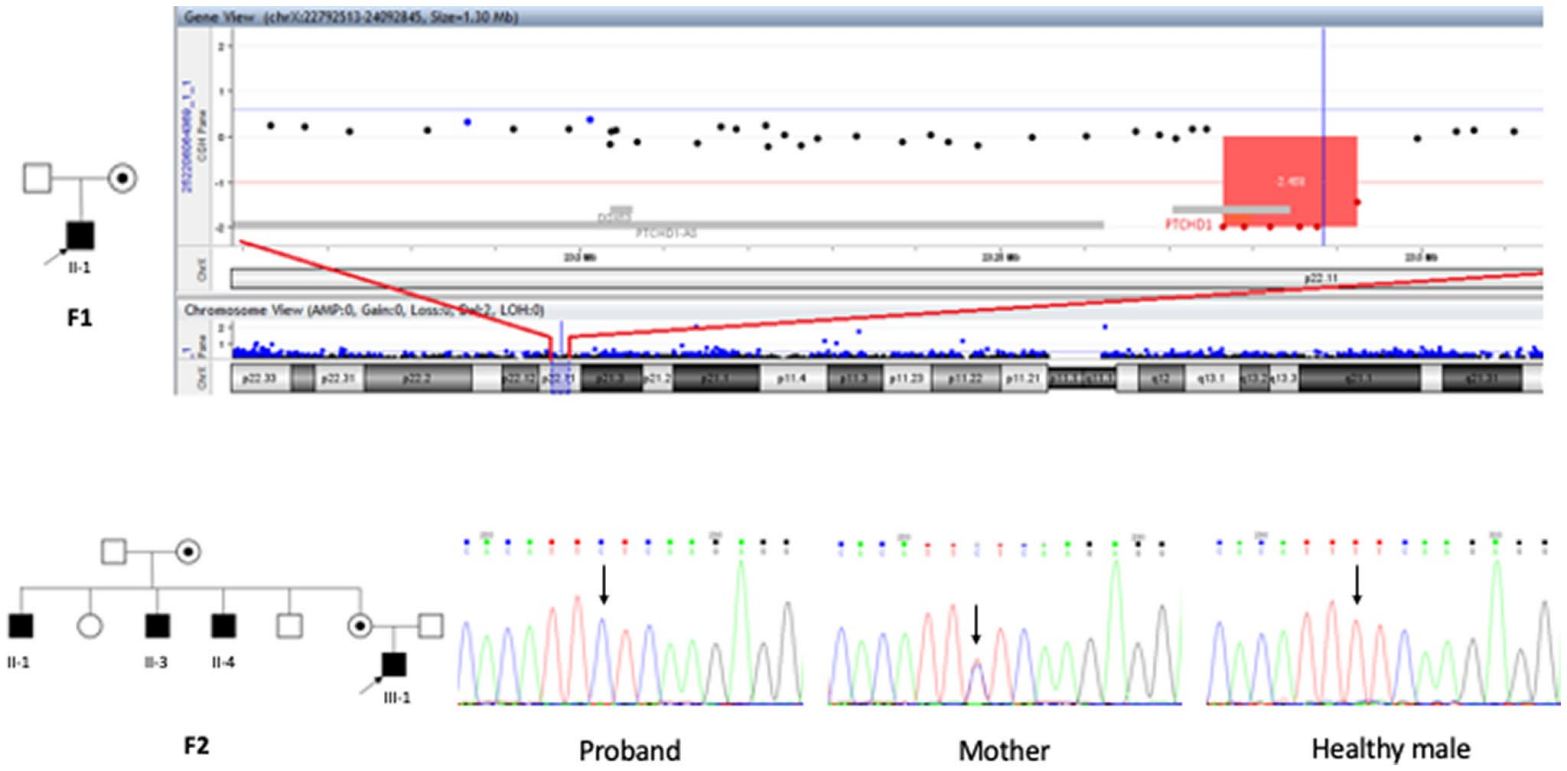
Pedigree and graphic representation of *PTCHD1* (NM_173495.3) variants. Upper panel: on the left, the pedigree of patient II 1 F1 (family 1); on the right, the graphic representation of the *PTCHD1* deletion breakpoints identified by array-CGH analysis. Lower panel: on the left, pedigree of family 2; on the right, electropherograms of the proband, carrier mother, and healthy control the c.1877 T > C transition.

Our cohort initially included five male individuals: one child with a *PTCHD1* microdeletion and another child along with his three maternal uncles with *PTCHD1* gene mutation (another uncle and an aunt of the second affected child were negative to direct Sanger sequencing and in good health as reported by family members) (Figure 1).

Unfortunately, one uncle with *PTCHD1* mutation did not consent to the neuropsychological and psychopathological assessment; therefore, our final cohort included only four affected individuals. All participants were recruited at the Child and Adolescent Psychiatry Unit and Genetics and Rare Diseases Research Division of the Bambino Gesù Children’s Hospital, Rome. The available information about patients’ medical history is reported in the following paragraphs. To guarantee privacy, each participant was identified by a code composed of an ordinal number indicating the family member [I, II, III and of the cardinal number family number (1 vs. 2)].

### 2.2 Patient II-1 F1 (family 1)

**Figure 2:**
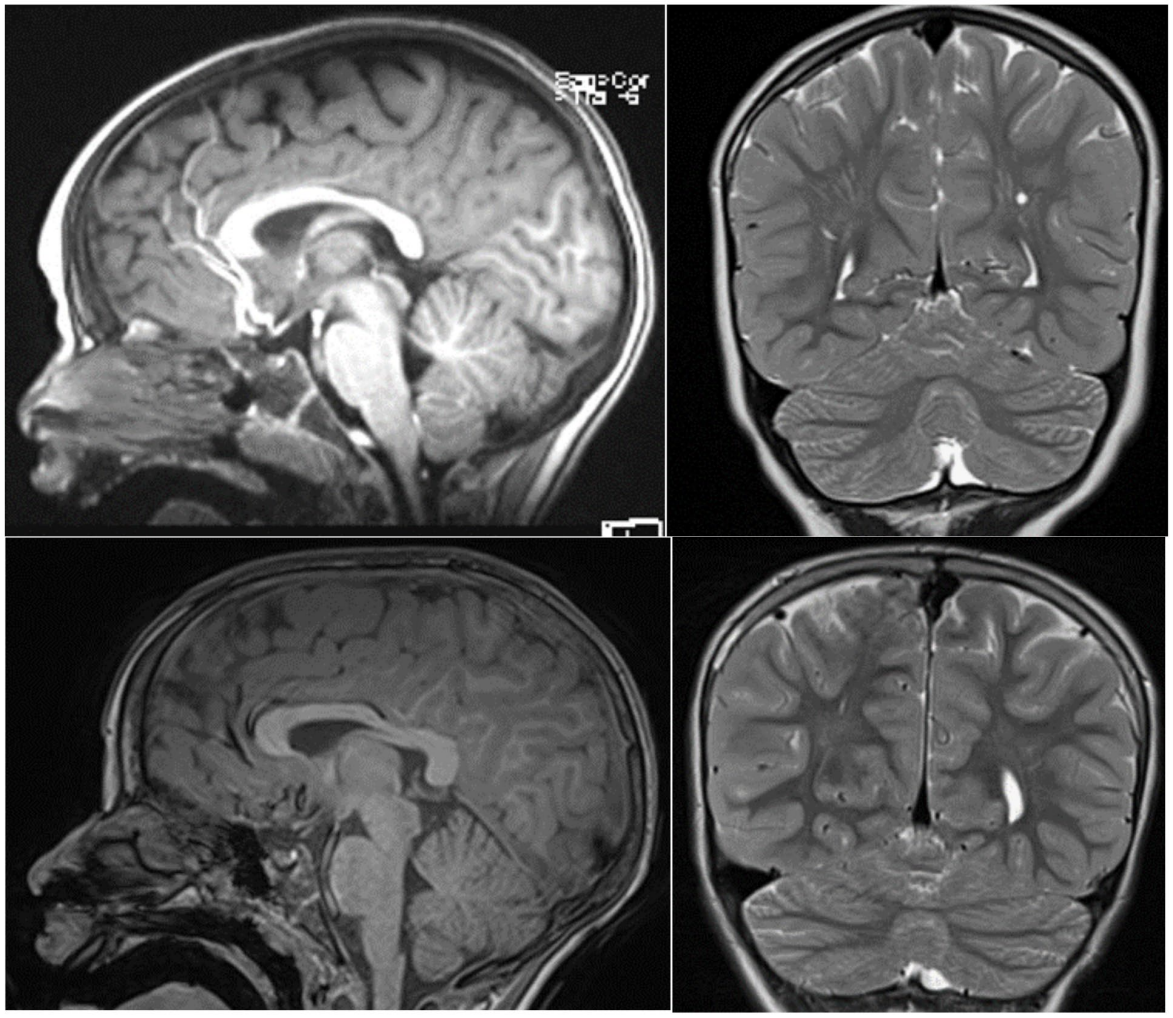
T2-midsagittal and coronal MRI neuroimages showing vermis cerebellar atrophy of patient II-1 F1 upper panel and patient III 1 F2 lower panel.

Patient II 1 F1 was seven at the time of the neuropsychological assessment. He was born at term through induced birth. APGAR score was 7–10. At birth, he weighed 2,450 gr, measured 52 cm in length, and had a cranial circumference of 34 cm. He reached the sitting position at 12 months, crawled at 19 months, and walked independently at 27 months. Psychomotor delay was first reported as early as the age of 3 months. Rehabilitation therapy started when he was 18 months old with a neuropsychomotor treatment and speech therapy at approximately 42 months old. Among the genetic conducted investigations, the analyses of the karyotype and the *FMR1* gene were normal. The array-CGH analysis showed (1) a microdeletion Xp22.11, extending approximately 80 Kb, involving the maternally inherited *PTCHD1* gene, and (2) a 5q21.2 microduplication, extending approximately 212 Kb and containing part of the *NUDT12* gene (absent in the mother, father not analyzable) which regulate the concentrations of individual nucleotides and of nucleotide ratios in response to changing circumstances. The geneticist evaluated duplication of the 5q21.2 region, even though the father was not analyzable, as not associated with known pathological conditions and is therefore of uncertain clinical significance. He was negative for the metabolic screening; the magnetic resonance imaging (*MRI*)–(Figure 2, upper panel)—and EEG in wakefulness and short sleep phase were normal. An odontostomatology examination showed malocclusion with increased overjet and atypical swallowing. No sleep problems were reported.

Patient II 1 F1 received a diagnosis of ID, ASD, and Mixed Emotions and Conduct Severe Disorder. Due to the behavior disorder, he commenced therapy with risperidone at the age of six. Currently, he undergoes physical, speech-language, and cognitive-behavioral therapy.

### 2.3 Patient III 1 F2 (family 2)

Patient III 1 F2 was eight at the time of the neuropsychological assessment. He was born at term following a regular pregnancy by dystocic vaginal delivery. At birth, he presented respiratory problems, necessitating transfer to the intensive care unit where he underwent resuscitation with a mask. An initial brain ultrasound revealed a normal ventricular system and parenchymal echodensity. He also presented with a right hydrocele. Maternal breastfeeding was prolonged, while weaning was regular. Sleeping–waking rhythm was reported as normal. At the time of our evaluation, sphincter control was not yet acquired. He achieved autonomous gait at 18 months and exhibited language delay (late talker). Orthoptic examination in 2015 was normal with ocular motility within limits. The audiometric examination was within normal limits, and brainstem auditory-evoked potentials showed an electrophysiological picture compatible with an auditory threshold within normal limits bilaterally. Parents reported a family history of developmental delay on the maternal side, with three of the mother’s siblings affected, while the other two were normal. He was negative for *FMR1* gene screening. Array-CGH at 1 Mb resolution was normal. *MRI* images were normal (Figure 2, lower panel). Patient III 1 F2 received the diagnosis of developmental delay with some atypical social features. He currently undertakes speech and psychomotor therapy.

### 2.4 Patient II 1 F2 (family 2)

Patient II 1 F2 was one of the three affected uncles of patient III 1 F2 and was 58 when he received our assessment. He personally reported his medical history, revealing that he was born at term following regular pregnancy. Autonomous gait was acquired at 18–24 months, while sphincter control was achieved at 5–6 years of age. He had acquired basic skills in reading and learned basic academic skills, and currently, he occasionally works as a laborer. He reported the presence of motor stereotypies or tics (with shoulders) when he was young that have decreased over time and the presence of a malformation of the little finger of the right hand. He denied any major medical disorders and currently lives with a sister and another brother. Upon neuropsychiatric examination, no focal deficits or disorders of content and thought form were observed. He received a diagnosis of mild–moderate ID in *PTCHD1* mutation.

### 2.5 Patient II 3 F2 (family 2)

Patient II 3 F2, one of the three affected uncles of patient III 1 F2, underwent our evaluation at the age of 53. The patient himself provided a detailed account of his medical history. He was born at term following a regular pregnancy and reported achieving autonomous gait between 18 and 24 months, with sphincter control established at 5–6 years of age. No major medical disorders were reported, but he did undergo surgeries for inguinal hernia and deviated nasal septum. Currently, he lives with his wife and twin sons, who are in apparent good health, and occasionally works as a laborer. During the neuropsychiatric examination, no focal deficits or disorders of content and thought form were observed. He received a diagnosis of mild–moderate ID in *PTCHD1* mutation.

### 2.6 Patient II 4 F2 (family 2)

Patient II 4 F2 was one of the three affected uncles of patient III 1 F2 and was 51 years old when he received the genetic assessment. Unfortunately, he was not available for neuropsychological and psychopathological evaluations; however, relatives reported that he was affected by ID.

### 2.7 Cytogenetics and molecular cytogenetics

#### 2.7.1 ARRAY-CGH analysis

Clinical data were obtained in accordance with the ethical standards of the review board of the Bambino Gesù Children Hospital (Rome, Italy). Informed consent was signed by the patient’s mother. DNA of the proband and his mother was isolated from peripheral blood using a QIAsymphony automatic extractor (QIAGEN, www.qiagen.com). Array-CGH analysis was performed on the patient’s DNA using an Agilent 4x180K oligo-array platform, according to the manufacturer’s instructions.^1^ Images were obtained using Agilent DNA Microarray Scanner and Agilent Scan Control Software (v A.8.4.1), while analyses were performed using Agilent CytoGenomics software (v 4.0.3.12). Confirmation and segregation tests on the patient’s and his mother’s DNA were performed by real-time PCR, with primers for *PTCHD1*, using a sybr green assay, as previously described (13).

The array-CGH analysis on the patient’s II 1 F1 DNA showed a microdeletion involving The short Arm of chromosome X, at Xp22.11, 80 kb In size, including exons 2 and 3 of *PTCHD1* (NM_173495). The real-time PCR analysis on The patient’s and His mother’s DNA revealed The maternal segregation of the deletion. Arr[hg19] Xp22.11(23,383,292 − 23,463,336) x0 mat (Figure 1 upper panel, pedigree, and graphical representation of array-CGH data). The array-CGH analysis on the patient’s III 1 F2 DNA was normal.

#### 2.7.2 Next-generation sequencing (NGS) and analysis to identify the *PTCHD1* variant in patient III 1 F2

Using genomic DNA, the exonic regions and flanking splice junctions of the genome were captured using a custom Agilent Sure Select Target Enrichment system XLID Panel including 114 genes.^2^ Sequencing was performed on an Illumina system with 100 bp or greater paired-end reads. The reads were aligned to human genome build GRCh37/UCSC hg19 and analyzed for sequence variants using a custom-developed analysis tool, and standard protocols were followed for variant interpretation.

#### 2.7.3 3D structure and multiple sequence alignment

**Figure 3:**
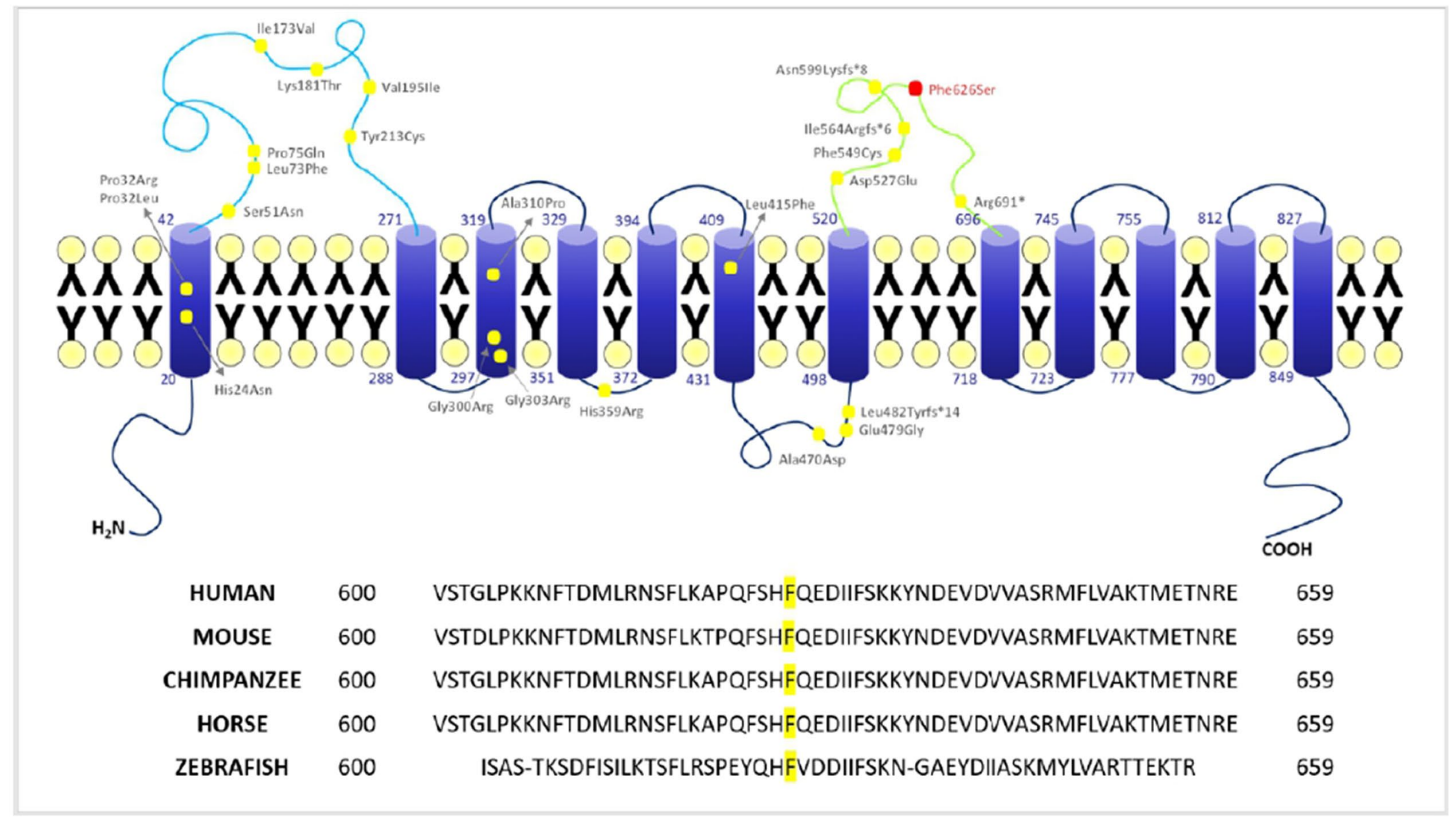
Localization of *PTCHD1* variants and conservation of F626 among different species. Upper panel: 2D structure of *PTCHD1* protein and localization of the missense variants identified to date. Lower panel: conservation of phenylalanine (F) 626 among different species (Clustal Omega).

**Figure 4:**
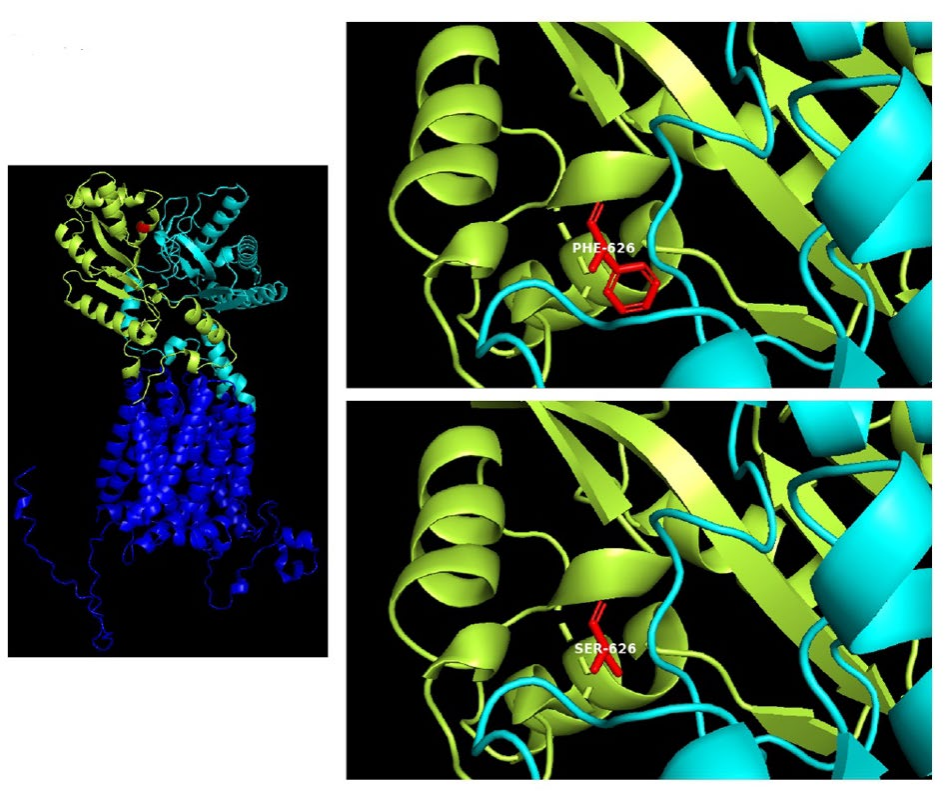
3D structure of the *PTCHD1* missense variant identified in the present study. Human patched domain-containing protein 1 (*PTCHD1*), 3D structure (Uniprot Q96NR3): extracellular domain 1 (aa: 43–270) in cian; extracellular domain 2 (aa: 521–695) containing the missense variant p.F626S, in limon.

The 3D structure is based on an AlphaFold computed structure model (CSM) of patched domain-containing protein 1 (AF_AFQ96NR3F1). The “per-residue confidence score” (pLDDT) of our residue (F626) is 88.34 and consequently classified as “Confident” (90 > pLDDT >70). Molecular structures were rendered with PyMOL (Figure 4). The multiple sequence alignment of *PTCHD1* protein among organisms around the sites of p.F626S was obtained with Clustal Omega^3^ (Figure 3).

### 2.8 Neuropsychological and psychopathological assessment

The assessment was conducted by a team of trained and specialized child psychiatrists, psychologists, and speech and language therapists and consisted of clinical observations, standardized evaluations, and eventually parent interviews. Tests were administered during routine clinical activities, usually lasting 3 working days. It is important to underline that participants underwent a heterogeneous assessment as tests were selected according to clinical purposes specific to each patient. Table 1 shows in detail each patient’s assessment. Cognitive profile and adaptive functioning are provided for all the subjects in the section results (Table 2).

**Table 1:** Detailed evaluation undertaken by the studied cohort.

Participant no code	Evaluation
II 1 F1	LEITER-3
	ABAS II
	TFL
	PinG PAROLE IN GIOCO
	CBCL 6–18
	CPRS-R: L
	ADOS 2
III 1 F2	WISC IV
	ABAS II
	VMI
	CBCL 6–18
	CCC-2
	SCQ-L
II 1 F2	LEITER 3
	ABAS II
	SCL-90-R
II 3 F2	LEITER 3
	ABAS II
	SCL-90-R

ABAS II, Adaptive Behavior Assessment System, Second Edition; ADOS 2, Autism Diagnostic Observation Schedule – 2nd Edition (ADOS-2); TFL, Phono-Vocabulary Test; PING, Picture Naming Game; CBCL 6–18, Children Behavior Check List 6–18; CPRS-R: L, Conners Parent Rating Scale-Revised: Long Version; WISC IV, Wechsler Intelligence Scale for Children-Fourth Edition; VMI, Visual-Motor Integration; CCC-2, Children’s Communication Checklist-2; SCQ-L, Social Communication Questionnaire-Lifetime version; SCL-90-R, Symptom Checklist-90-Revised; F1 Family 1; F2 Family 2.

**Table 2:** Cognitive and adaptive functioning of studied cohort.

Cognitive and adaptive functioning	Patient II 1 F1	Patient III 1 F2	Patient II 1 F2	Patient II 3 F2	M ± SD
IQ	64	74*	45	49	58 ± 13
ABAS II – GAC score	59	73	64	57	63 ± 7
ABAS II – Conceptual	<57	69	57	61	61 ± 6
ABAS II – Social	75	78	62	66	70 ± 7
ABAS II – Practical	63	78	66	53	65 ± 10

*M*, mean; SD, standard deviation; IQ, intelligence quotient; *Perceptual reasoning index; GAC, general adaptive composite; F1, Family 1, F2 Family 2.

#### 2.8.1 Cognitive assessment

Cognitive profile was preferentially assessed through the cognitive battery of the Leiter International Performance Scale-Third Edition (Leiter-3) (14), which is administered to subjects with clear speech impairment. Just one boy (patient III 1 F2) was evaluated through the Wechsler Intelligence Scale for Children-Fourth Edition (WISC-IV) (15).

*Leiter-3* allows for the assessment of non-verbal cognitive, attentional, and neuropsychological abilities, designed to be administered to people from 3 to +75 years. It provides a non-verbal intelligence quotient (NVIQ; herein after IQ, intelligence quotient), as well as percentile and age-equivalent scores for each subtest.

*WISC-IV* is a measure of intellectual performance of individuals ranging from 6 years to 16 years and 11 months. It provides a Full-Scale IQ corresponding to the overall level of intelligence together with four main reasoning indices (Verbal Comprehension VCI, Perceptual Reasoning PRI, Working Memory WMI, and Processing Speed PSI). In our study, PRI has been used to compare the individuals assessed through WISC-IV to the remaining subjects’ IQs.

#### 2.8.2 Adaptive behavior assessment

Adaptive functioning has been assessed using the *Adaptive Behavior Assessment System*, Second Edition (ABAS-II) (16). The ABAS-II is a comprehensive checklist covering a broad spectrum of skill areas related to development, behavior, and cognitive abilities, spanning the entire lifespan from early infancy to adulthood. The assessment can be completed by parents or other informants, and age-appropriate versions are available. The ABAS-II encompasses subscales for communication, community use, functional academics, home living, health and safety, leisure, self-care, self-direction, social, and work. Composite scores, including the general adaptive composite (GAC), conceptual, social, and practical, are derived from the sum of the scaled scores. These composite scores have a mean of 100 and a standard deviation of 15.

#### 2.8.3 Language assessment

Language skill assessment has been carried out only with patient II 1 F1, by using the following tests:

1. *Picture Naming Game (PiNG) test*, which measures lexical comprehension and production in Italian toddlers in the age group of 19–37 months (17). The PiNG test consists of four main blocks: Noun Comprehension, Noun Production, Predicate Comprehension, and Predicate Production. The total score is converted into Lexical Quotient (LQ).
2. The *Phono-Vocabulary Test (Test Fonolessicale—TFL)* assesses receptive and expressive vocabulary in children from 2, 5 up to 6 years of age (18). The test includes 45 tables with 4 images each: a target, a phonological distractor, a semantic distractor, and a non-related distractor. The examiner pronounces a word illustrating one of the four pictures on the table and asks the participant to choose the picture(s) that better corresponds to the pronounced word. The total score is converted into percentiles (pc).

#### 2.8.4 Visuomotor integration assessment

Visuomotor integration has been assessed only in patient III 1 F2 through the *Developmental Test of Visual-Motor Integration*, Sixth Edition (VMI). VMI is a standardized, norm-referenced assessment involving copying geometric forms to determine the visuomotor integration in children and adolescents from 3 years up to 18 years of age (19).

#### 2.8.5 Psychopathological assessment

##### 2.8.5.1 Autism diagnostic observation schedule second edition (ADOS-2)

The ADOS-2 (20) has been administered to patient II 1 F1 to investigate the presence of autistic symptoms. It can be used to evaluate almost anyone suspected of having autism spectrum disorder (ASD), as young as 12 months old with language disorders to verbally fluent adults. It measures four main domains: communication, social interaction, play, and restricted and repetitive behaviors. Scores for these domains are summed, and the total score is compared to thresholds resulting in an ADOS classification of autism, autism spectrum, or non-spectrum.

##### 2.8.5.2 Children behavior check list (CBCL) 6–18

CBCL 6–18 has been administered to participant patient II 1 F1 and patient III 1 F2 to assess their behavioral profile (21). The CBCL 6–18 consists of eight syndrome scores: Anxious/Depressed, Withdrawn/Depressed, Somatic Problems, Social Problems, Thought Problems, Attention Problems, Rule Breaking, and Aggressive Behavior. Furthermore, it includes other scales, namely, the Competence Scale, Internalizing, Externalizing, and Other Problems Scales, DSM-Oriented Scales, and 2007 Scales. According to the ASEBA Assessment Data Manager (ADM), the t-scores of the Syndrome Scales, DSM-Oriented Scales, and 2007 Scales from 67 to 70 are in the borderline range, while the t-scores above 70 are in the clinical range; concerning the Internalizing, Externalizing, and Other Problems Scales, the t-scores of 60 to 63 fall in the borderline range, while the t-scores above 63 fall in the clinical range.

##### 2.8.5.3 Conners parent rating scale-revised: long version (CPRS-R: L)

Child behavior was evaluated using the Conners Parent Rating Scales-long version (CPRS-R: L) (22), which is designed to address the need for a multimodal assessment of children and adolescents’ behavioral difficulties. The following seven subscales are included: Cognitive Problems (CPs), Oppositional (O), Hyperactivity-Impulsivity (H-I), Anxious-Shy (A-S), Perfectionism (P), Social Problems (SP), and Psychosomatic (P). Additionally, different indexes, such as a global index, an attention deficit hiperactivity disorder (ADHD) index, and a DSM–IV–TR-related disorder index, are provided. The CPRS-R: L is administered to children aged from 3 up to 17 years, and parents rate each item on a Likert scale from 0 (not true at all) to 3 (very much true). Raw scores are converted into t-scores and percentile scores. Significant scores range from a low t-score of 61 (mildly atypical) to above 70 (markedly atypical) (23).

##### 2.8.5.4 Social communication questionnaire-lifetime version (SCQ-L)

The SCQ-L (24) is a brief parent-report screening test developed to evaluate communication skills and social functioning in children with a suspected diagnosis of autism or autism spectrum disorder (ASD). Each item in the SCQ requires a dichotomous “yes”/ “no” response, receiving a value of 1 point for abnormal behavior and 0 points for the absence of abnormal behavior. The SCQ-L does not aid in providing a diagnosis, but scores above the cutoff of 15 suggest that the individual is likely to be on the autism spectrum and that the person should be referred for a more complete evaluation.

##### 2.8.5.5 Children’s communication checklist-2 (CCC-2)

The CCC-2 test is a parent or caregiver rating scale that includes two main sections: language and pragmatics. The language domain consists of four main subscales: Speech, Syntax, Semantics, and Coherence. Six subscales are included in the Pragmatic domain: Initiation, Scripted Language, Context, Nonverbal Communication, Social Relations, and Interests. Scaled scores may be derived from each subscale’s total raw score. Scaled scores of four or lower on a subscale indicate impairment within that area, while a scaled score of six or higher indicates typical functioning. CCC-2 can be administered to people aged between 4 and 11 years old and is helpful to detect if a person needs a deeper evaluation (25).

##### 2.8.5.6 Symptom checklist-90-revised (SCL-90-R)

The SCL-90-R aims to evaluate a broad range of psychological problems in people aged 13 or older (26). It is a 90-item questionnaire, which measures nine main symptom constructs: Somatization, Obsessive Compulsive Disorder, Interpersonal Sensitivity, Depression, Anxiety, Hostility, Phobic Anxiety, Paranoid Ideation, and Psychoticism. Each item has five following response categories: 0 = Not at all, 1 = little, 2 = some, 3 = very, and 4 = severe. The scores on each dimension are means of the scores of all items of the construct. Furthermore, the Global Severity Index can be used as a summary of the test. To suit clinical work, the SCL-90-R scores are converted to standard t-scores (normal range = t-score between 40 and 60).

## 3 Results

### 3.1 Next-generation sequencing (NGS) and analysis

The *PTCHD1* variants are named according to Refseq NM_173495.3, which encodes a protein of 888 amino acids (Refseq NP_775766.2). The potential effects of the missense variant were predicted using SIFT [(27), https://sift.bii.a-star.edu.sg/] and Polyphen-2 [(28), http://genetics.bwh.harvard.edu/pph2/], and protein sequences were aligned with Clustal Omega (29). The presence of the variant in the general population was assessed using the gnomAD database (http://gnomad.broadinstitute.org/, v2.1.1). The variant was confirmed by direct Sanger sequencing in the patient and family members. A maternally derived c.1877 T > C: p. Phe626Ser *PTCHD1* variant has not been reported in the Human Gene Mutation Database, or any public SNP database(s) was identified. The variant co-maternally inherited *PTCHD1* with the disease in all three affected maternal uncles (Figure 1, lower panel with Pedigree and chromatograms).

### 3.2 3D structure and multiple sequence alignment

The T to C transition results in an amino acid substitution from Phe to Ser at residue 626, which is located within a large loop predicted to face the cytosol (extracellular domain 2). As shown in Figure 4, amino acids surrounding this phenylalanine are partially conserved in the PTCHD family of proteins and PTCHD2 also shows the same residue (F) at position 626, suggesting that this area is most likely to be functionally important. Phenylalanine is an aromatic, hydrophobic, amino acid while serine is a slightly polar, amino acid. Therefore, it can be expected that the disavowable F to S substitution might interfere with the extracellular domain function, and hence with *PTCHD1* interaction with other proteins.

### 3.3 Cognitive and adaptive functioning

As the cognitive, adaptive, and psychopathological evaluations were heterogeneous, we report two main sections in this study: one, describing the cognitive and adaptive functioning of the whole study cohort, and a second one, providing a descriptive analysis of the assessment differentially undertaken by each patient.

#### 3.3.1 Whole study cohort

As Table 2 exhibits, all subjects were characterized by a low mean IQ score (*M* = 58, SD = 13), with the two adults (patient II 1 F2 e II 3 F2) performing worse than the children. Furthermore, the results on adaptive behavior measured with GAC showed an overall low functioning (*M* = 63; SD = 7), with social adaptation being averagely higher than conceptual one. Cognitive and adaptive profiles of patient III 1 F2 who was the only patient evaluated with WISC-IV were globally more preserved if compared with the ones of the remaining study cohort.

#### 3.3.2 Descriptive analysis of each patient’s assessment

##### 3.3.2.1 Patient III 1 F2

Table 3 shows in detail the results of the evaluation undertaken by patient II 1 F1. Regarding the CBCL Subscales, scores reached the clinical significance on Activities, School, and Total Competence Scales. Concerning Syndrome Scales, the only clinical symptom scores were recorded on Thought and Attention problems scales. The remaining scores fell in the non-clinical level, with only the aggressive behavior subscale being in the borderline range. Concerning the Internalizing, Externalizing, and Total problems scales, only Total problems were over the clinical cutoff, with Internalizing and Externalizing Problems falling in the at-risk range. Additionally, only attention-deficit/hyperactivity DSM IV-oriented scale scores reached clinical significance. The remaining subscales were in the normal range.

**Table 3:** Psychopathological features and language skills of patient II 1 F1.

CBCL subscales		Score
Competence scales	Activities	23
	Social	37
	School	**25**
	Total competence	**20**
Syndrome scales	Anxious/depressed	59
	Withdrawn/depressed	62
	Somatic complaints	61
	Social problems	65
	Thought problems	**70**
	Attention problems	**75**
	Rule-breaking behavior	53
	Aggressive behavior	*68*
Internalizing, externalizing, and total problems scales	Internalizing	*63*
	Externalizing	*65*
	Total Problems	**70**
DSM IV-oriented scales	Depressive problems	65
	Anxiety problems	60
	Somatic problems	50
	Attention deficit/hyperactivity	**72**
	Oppositional defiant	66
	Conduct problems	60
2007 Scales	Sluggish cognitive tempo	63
	Obsessive-compulsive problems	55
	Stress problems	66

CPRS-R:L subscales	Score
A Oppositional	52
B Cognitive problems/inattention	*65*
C Hyperactivity	53
D Anxious-shyness	53
E Perfection	46
F Social problems	43
G Psychosomatic	43
H Conners’ ADHD index	*63*
I Conners’ Global Index Restless-Impulsive	61
J Conners’ Global Index, Emotional Lability	61
K Conners’ Global Index, Total	63
L DSM IV Symptom Subscales, Inattentive	59
M DSM IV Symptom Subscales, Hyperactive/Impulsive	53
N DSM IV Symptom Subscales, Total	56

ADOS-2	Score
Affective-social	11
Restricted and repetitive behaviors	4
Total	**15**
Language skills	Qualitative analysis
TFL	Below average
PinG	Below average

CBCL 6–18, Children Behavior Check List 6–18; CPRS-R: L, Conners Parent Rating Scale Revised: Long Version; TFL, Phono-Vocabulary Test; PinG, Picture Naming Game; below average, for chronological age. Bold = clinical; Italic = borderline. ADOS-2 cutoff ASD diagnosis = 6.

Concerning CPRS-R: L, all the scores were below the conventional threshold, with only Cognitive problems/Inattention and Conners’ Global Index Restless-Impulsive approaching conventional levels of significance.

Finally, regarding language skills, lexical comprehension and production and morphosyntactic comprehension scores corresponded to the ones in the range of a mental age of 3 years.

##### 3.3.2.2 Patient III 1 F2

Taking into account the CBCL 6–18, scores reached the significance on the Activities, Social, and Total Competence Scale, while concerning the CBCL Syndrome scales only withdrawn/depressed were in the clinical range, with Anxious/Depressed and Thought problems approaching but not reaching significance. Concerning Internalizing, Externalizing, and Total problems scales, only Internalizing problems and Total problems scales scores fell in the significant range, while considering DSM IV-oriented scales only Anxiety problems were within the conventional bounds of statistical significance. The stress problems subscale was in the clinical range as well. The remaining scores did not reach significance.

Taking heed of SCQ, the scores exceeded the conventional cutoff, signaling the presence of socio-relational difficulties. This result was in line with the CCC-2 scores, the majority of which fell in the clinical range (see Table 4 for more details). Finally visuomotor integration abilities evaluated with VMI were lower than the chronological age.

**Table 4:** Psychopathological features and visuomotor and language skills of patient III 1 F2.

CBCL subscales		Score
Competence scales	Activities	**25**
	Social	**29**
	School	40
	Total competence	**23**
Syndrome scales	Anxious/depressed	*69*
	Withdrawn/depressed	76
	Somatic complaints	61
	Social problems	56
	Thought problems	*67*
	Attention problems	55
	Rule-breaking behavior	57
	Aggressive behavior	61
Internalizing, externalizing, and total problems scales	Internalizing	**71**
	Externalizing	60
	Total Problems	**65**
DSM IV-oriented scales	Depressive problems	63
	Anxiety problems	**70**
	Somatic problems	50
	Attention deficit/hyperactivity	62
	Oppositional defiant	52
	Conduct problems	51
2007 Scales	Sluggish cognitive tempo	50
	Obsessive-compulsive problems	64
	Stress problems	**72**
VMI	Visuomotor integration visual	**<45**
	Motor	62
	Total	71
SCQ		**Score**
*Subscales*	Total	16
	Social interaction	5
	Communication	7
	Stereotypical behavior	3

CCC-2 subscales	Score
A Speech	**4**
B Syntax	**3**
C Semantics	1
D Coherence	9
E Inappropriate initiation	*5*
F Stereotyped language	*6*
G Context	3
H Non-verbal communication	*4*
I Social Relations	*5*
L Interests	4
GCC	**38**
SIDC	-2

CBCL 6–18, Children Behavior Check List 6–18; VMI, Visual-Motor Integration; SCQ, Social Communication Questionnaire-Lifetime version; CCC-2, Children’s Communication Checklist-2; GCC, General Communication Composite; SIDC, Social Interaction Difference Index. Bold = clinical; Italic = borderline. SCQ cutoff = 15. GCC CC2 – cutoff = 55.

##### 3.3.2.3 Patient II 1 F2 and patient II 3 F2

As the only two adult patients both filled the SCL-90-R, the results are presented together in Table 5. All SCL-90-R rating categories resulted in the clinical range for both patients, underlining a global elevated symptom severity.

**Table 5:** SCL-90-R scores.

	SOM	O-C	I-S	DEP	ANX	HOS	PHOB	PAR	PSY	GSI	PSDI
Patient II 1 F2	**>75**	**>75**	**>75**	**>75**	**>75**	**>75**	**>75**	**>75**	**>75**	**>75**	*69–70*
Patient II 3 F2	**>75**	**>75**	**>75**	**>75**	**>75**	**72**	**>75**	*67*	**74**	**>75**	*61–62*

SCL-90-R, symptom checklist-90-revision; SOM, somatization; O-C, obsessive-compulsive; I-S, interpersonal sensitivity; DEP, depression; ANX, anxiety; HOS, hostility; PHOB, phobic anxiety; PAR, paranoid ideation; PSY, psychoticism; GSI, global severity index; PSDI, positive symptom distress index; PST, positive symptom total. Composite. Bold = clinical; Italic = borderline. F1, Family 1, F2 Family 2.

## 4 Discussion

More than 70 deleterious genomic microdeletions or truncating mutations of the X-linked gene *PTCHD1* have been involved in neurodevelopmental disorders with ID and/or ASD. Non-synonymous variants were also identified and recently showed to impair *PTCHD1* N-glycosylation and reduce protein stability (TM1 P32R and TM3 G303R) (29) while other variants (Ectodomain 1 (ECD1) Lys181ThrCys and Tyr 213Cys) and TM domain (Pro32L, Gly300Arg, Ala310 Pro) were found to determine a weak membrane localization for the *PTCHD1* protein consequent to its retention in the ER (30). The novel variant p. Phe626Ser is located in the second ectodomain (ECD2) of the protein. The phenylalanine residue is highly conserved among species as the surrounding amino acids, suggesting that this area is most likely to be functionally important (Figure 3 lower panel, showing amino acid conservation of the phenylalanine 626 residue). Of the nine variants found in the second ectodomain, seven are truncating variants, while two are non-synonymous (Phe549Cys and Asp527Glu). No functional studies have been performed to determine the impact of these variants on *PTCHD1* function. Recent research indicates that, unlike PTCH1, *PTCHD1* ectodomains do not bind SHH, suggesting that *PTCHD1* has not a role as canonical SHH inhibitor, instead its interaction with proteins involved in cell stress response and RNA granule formation [already implicated in ID and/or ASD such as DYRK1A (31, 32)]. In the human brain, *PTCHD1* is preferentially expressed in the cerebellum, cortex, and temporal lobe, which represent important areas for high-level cognitive functions (33). *PTCHD1* is localized in the dendritic spines and can interact with postsynaptic scaffolds PSD95 and SAP102, protein coded by DGL3 responsible for a form of ID X-linked (34). Its deletions or mutations can give rise to a broad spectrum of neurodevelopmental difficulties. Previous studies (1–3, 35) have already documented ID, ASD, global developmental delay, infantile hypotonia, and motor incoordination in individuals with *PTCHD1* microdeletions or mutations. Some patients may also exhibit aggressive behavior, sleep disorders, ADHD, and other psychiatric issues (1, 36, 37) suggesting *PTCHD1*’s possible involvement in overlapping phenotypes in the spectrum of intellectual, neurodevelopmental, and autism disorders. With the aim to further contribute to the cognitive – behavioral phenotyping of *PTCHD1* disorders, in this study, we report the neuropsychological and psychopathological profiles of four patients, providing quantitative data from standardized evaluations. Our analysis shows that only one patient out of four (patient II 1 F1) presented a history of suffering at birth. *MRI* of the two children (patient II 1 F1 and III 1 F2) did not reveal in any case intracranial pathological features neither acute nor morphostructural. In our cohort, we found no peculiar dimorphisms, major congenital abnormalities, or association with epilepsy. On the other hand, all patients presented a moderate-to-severe delay in psychomotor development, with a history of motor coordination deficit in the case of the child without ASD. Motor stereotypies were present in the entire study cohort. Additionally, our clinical results showed that the IQs of the two adults (patients II 1 F2 and II 3 F2) were significantly lower than IQs of the two children (patients II 1 F1 and III 1 F2), maybe suggesting a cognitive decline with age in people with *PTCHD1* disorders, even though longitudinal studies are required to draw this conclusion. Additionally, in accordance with studies showing that ID is more severe in people with a concurrent diagnosis of ASD, patient III 1 F2, who did not have an ASD comorbidity, exhibited the least impaired IQ. Interestingly, neither patient II 1 F2 nor patient II 3 F2 exhibited autistic-like symptomatology. Contrastively to IQ differences with age, we did not observe any difference in adaptive functioning comparing “childhood” to “adulthood.” Even though the analysis of only four patients is not enough to confirm any hypothesis, we can speculate that this finding may be explained by the fact that adaptive behavior is usually more preserved than cognitive functioning also in people with ID (38). Regarding the psychopathological features, patient II 1 F1 presented thought problems, social and attention disorders in association with a strong language impairment. More specifically, analyzing the CBCL 6–18 subscales, the scores reached the clinical significance on Activities, School, and Total Competence Scales. Concerning Syndrome Scales, the only clinical symptom scores were recorded on Thought and Attention problems scales. The remaining scores fell in the non-clinical level, with only aggressive behavior subscale being in the borderline range. Internalizing and Externalizing Problems score fell in the at-risk range, which maybe suggesting a neurodevelopmental trajectory at risk for disruptive behavioral disorder or psychosis. Attention deficit/hyperactivity DSM IV-oriented scale scores reached clinical significance. He presented also reduced language performances, with comprehension, and production scores in the range of a mental age of 3 years. Patient III 1 F2 showed significant internalizing disorders, associated with anxiety and social problems, as revealed by CBCL 6–18. SCQ and CCC-2 scores were above the cutoff as well. These data are important as this patient, even though exhibiting socio-communicative deficits, did not fully meet criteria for a diagnosis of ASD according to DSM 5. Visuomotor deficits were also depicted. When comparing the two children (patient II 1 F1 and patient III 1 F2), the first patient, who was a carrier of a microdeletion, presented a more severe clinical phenotype with a codified diagnosis of ASD associated with cognitive impairment, and a behavioral disorder such that he has been on antipsychotic drug therapy since the age of six. Finally, the two adults (patients II 1 F2 and II 3 F2) presented clinical scores in all the sub-areas investigated by the SCL-90, which maybe suggesting an increase of psychopathological problems with age. Even though dysfunction of the thalamic reticular nucleus induced by *PTCHD1* and consequent sleep disorders have been reported in the literature (37), we did not observe any sleep disorder in our sample; on the other hand, we must consider that the number of patients that we analyzed is too small to make any inference.

Although more cases and longitudinal studies are required, our report underlines that *PTCHD1* mutations or microdeletions are associated with a co-occurrence of cognitive and psychological disorders that may worsen ID severity. This result poses an indication for early cognitive and occupational enhancement interventions for patients with *PTCHD1* mutations or deletions. A through follow-up is therefore necessary to define the best strategies for the rehabilitative processes management and for the analytical study of the neurodevelopmental trajectories.

## Data availability statement

The datasets for this article are not publicly available due to concerns regarding participant/patient anonymity. Requests to access the datasets should be directed to the corresponding author.

## Ethics statement

The studies involving humans were approved by Informed consent was obtained from all parents prior to participation and after receiving a comprehensive description of the study. The study was performed in accordance with the Declaration of Helsinki (1964), as revised in 2008, and was approved by the Institutional Review Board of Bambino Gesù Children’s Hospital. The studies were conducted in accordance with the local legislation and institutional requirements. Written informed consent for participation in this study was provided by the participants’ legal guardians/next of kin. Written informed consent was obtained from the individual(s), and minor(s)’ legal guardian/next of kin, for the publication of any potentially identifiable images or data included in this article.

## Author contributions

FAMM: Data curation, Formal analysis, Investigation, Writing – original draft, Writing – review & editing. AM: Investigation, Methodology, Writing – original draft. VA: Visualization, Writing – review & editing. ChS: Visualization, Writing – review & editing. DS: Validation, Visualization, Writing – review & editing. CiS: Validation, Visualization, Writing – review & editing. MF: Visualization, Writing – review & editing. GP: Data curation, Visualization, Writing – review & editing. AN: Visualization, Writing – review & editing. GZ: Data curation, Methodology, Validation, Writing – original draft. MD: Validation, Visualization, Writing – review & editing. SV: Supervision, Visualization, Writing – review & editing. PA: Conceptualization, Data curation, Supervision, Validation, Visualization, Writing – original draft, Writing – review & editing.

## Footnotes

^1^ www.agilent.com

^2^ https://ggc.org/test-finder-item/x-linked-intellectual-disability-xlid-sequencing-panel

^3^ https://www.ebi.ac.uk/Tools/msa/clustalo/
