# Corrigendum: PTCHD1 gene mutation/deletion: the cognitive-behavioral phenotyping of four case reports

**DOI:** 10.3389/fpsyt.2024.1375954

**Published:** 2024-02-13

**Authors:** Federica Alice Maria Montanaro, Alessandra Mandarino, Viola Alesi, Charles Schwartz, Daniela Judith Claps Sepulveda, Cindy Skinner, Michael Friez, Gabriele Piccolo, Antonio Novelli, Ginevra Zanni, Maria Lisa Dentici, Stefano Vicari, Paolo Alfieri

**Affiliations:** ^1^ Child and Adolescent Neuropsychiatry Unit, Department of Neuroscience, Bambino Gesù Children’s Hospital, IRCCS, Rome, Italy; ^2^ Laboratory of Medical Genetics, Translational Cytogenomics Research Unit, Bambino Gesù Children Hospital, IRCCS, Rome, Italy; ^3^ Department of Pediatrics and Human Development, College of Human Medicine, Michigan State University, East Lansing, MI, United States; ^4^ Neurology Unit, Bambino Gesù Children’s Hospital, IRCCS, Rome, Italy; ^5^ Greenwood Genetic Center, Gregor Mendel Circle, Greenwood, SC, United States; ^6^ Unit of Muscular and Neurodegenerative Disorders, Unit of Developmental Neurology, Bambino Gesù Children’s Hospital, IRCCS, Rome, Italy; ^7^ Genetics and Rare Diseases Research Division, Bambino Gesù Children’s Hospital, IRCCS, Rome, Italy

**Keywords:** *PTCHD1* gene, intellectual disability, autism spectrum disorder, rare genetic syndrome, cognitive-behavioral phenotype

**Text Correction**

In the published article, there was an error in the name of a Figure.

A correction has been made to the section “2.7 Cytogenetics and molecular cytogenetics”, subsection “2.7.1 ARRAY-CGH analysis”. This sentence previously stated:

“(**Figure 3** upper panel, pedigree, and graphical representation of array-CGH data).”

The corrected sentence appears below:

“(**Figure 1** upper panel, pedigree, and graphical representation of array-CGH data)”.

In the published article, there was an error in the name of another Figure.

A correction has been made to the section “2.7 Cytogenetics and molecular cytogenetics”, subsection “2.7.3 3D structure and multiple sequence alignment”. This sentence previously stated:

“The multiple sequence alignment of *PTCHD1* protein among organisms around the sites of p.F626S was obtained with Clustal Omega3 (**Figure 4** )”

The corrected sentence appears below:

“The multiple sequence alignment of PTCHD1 protein among organisms around the sites of p.F626S was obtained with Clustal Omega3 (**Figure 3** )”

In the published article, there was an error in a sentence.

A correction has been made to the section “2.7 Cytogenetics and molecular cytogenetics”, subsection “2.7.3 3D structure and multiple sequence alignment”. This sentence previously stated:

“Molecular structures were rendered with PyMOL.”

The corrected sentence appears below:

“Molecular structures were rendered with PyMOL (**Figure 4**).”

In the published article, there was an error in the reference to a Figure.

A correction has been made to the section “Discussion”. This sentence previously stated:

“(**Figure 4** lower panel, showing amino acid conservation of the phenylalanine 626 residue).”

The corrected sentence appears below:

“(**Figure 3** lower panel, showing amino acid conservation of the phenylalanine 626 residue).”

The authors apologize for this error and state that this does not change the scientific conclusions of the article in any way. The original article has been updated.

